# Mobilization of hematopoietic stem cells with the novel CXCR4 antagonist POL6326 (balixafortide) in healthy volunteers—results of a dose escalation trial

**DOI:** 10.1186/s12967-016-1107-2

**Published:** 2017-01-03

**Authors:** Darja Karpova, Susanne Bräuninger, Eliza Wiercinska, Ariane Krämer, Belinda Stock, Jochen Graff, Hans Martin, Achim Wach, Christophe Escot, Garry Douglas, Barbara Romagnoli, Eric Chevalier, Klaus Dembowski, Leon Hooftman, Halvard Bonig

**Affiliations:** 1 German Red Cross Blood Service BaWüHe, Institute Frankfurt, Frankfurt, Germany; 2 Clinical Trial Center Rhein-Main (KSRM), Pharmazentrum Frankfurt/ZAFES, Institute of Clinical Pharmacology, Goethe University, Frankfurt, Germany; 3 Department of Medicine II, Goethe University, Frankfurt, Germany; 4 Polyphor Ltd, Allschwil, Switzerland; 5 Institute for Transfusion Medicine and Immunohematology, Goethe University, Sandhofstr. 1, 60528 Frankfurt, Germany; 6 Department of Medicine, Division of Hematology, University of Washington, Seattle, WA USA; 7 Department of Internal Medicine, Division of Oncology, Section of Stem Cell Biology, Washington University Medical School, St. Louis, MO USA

**Keywords:** PEM-technology, CXCR4, Mobilization, Transplantation, Apheresis, Stem cell, Plerixafor, G-CSF, Clinical trial, Plasmacytoid dendritic cell

## Abstract

**Background:**

Certain disadvantages of the standard hematopoietic stem and progenitor cell (HSPC) mobilizing agent G-CSF fuel the quest for alternatives. We herein report results of a Phase I dose escalation trial comparing mobilization with a peptidic CXCR4 antagonist POL6326 (balixafortide) vs. G-CSF.

**Methods:**

Healthy male volunteer donors with a documented average mobilization response to G-CSF received, following ≥6 weeks wash-out, a 1–2 h infusion of 500–2500 µg/kg of balixafortide. Safety, tolerability, pharmacokinetics and pharmacodynamics were assessed.

**Results:**

Balixafortide was well tolerated and rated favorably over G-CSF by subjects. At all doses tested balixafortide mobilized HSPC. In the dose range between 1500 and 2500 µg/kg mobilization was similar, reaching 38.2 ± 2.8 CD34 + cells/µL (mean ± SEM). Balixafortide caused mixed leukocytosis in the mid-20 K/µL range. B-lymphocytosis was more pronounced, whereas neutrophilia and monocytosis were markedly less accentuated with balixafortide compared to G-CSF. At the 24 h time point, leukocytes had largely normalized.

**Conclusions:**

Balixafortide is safe, well tolerated, and induces efficient mobilization of HSPCs in healthy male volunteers. Based on experience with current apheresis technology, the observed mobilization at doses ≥1500 µg/kg of balixafortide is predicted to yield in a single apheresis a standard dose of 4× 10E6 CD34+ cells/kg from most individuals donating for an approximately weight-matched recipient. Exploration of alternative dosing regimens may provide even higher mobilization responses.

*Trial Registration* European Medicines Agency (EudraCT-Nr. 2011-003316-23) and *clinicaltrials.gov* (NCT01841476)

## Background

Most autologous and 80% of allogeneic hematopoietic stem cell transplantations (HSCT) are currently performed with mobilized peripheral blood stem cells [1]. These can be extracted by apheresis from the circulation after pretreatment of donors with the cytokine G-CSF, currently the most commonly used mobilizing agent [1]. Optimal mobilization with G-CSF in donors is relatively inconvenient as it takes 4–5 days [2–4]. G-CSF treatment, although generally considered safe, is regularly associated with acute (bone pain, flu-like symptoms, lethargy [5–8]) and more protracted (BM disruption [9], suppression of B-lymphopoiesis [10]) adverse events; moreover, a considerable list of contra-indications to G-CSF has been identified over the years, mostly related to the induction of neutrophila and neutrophil activation, but also to activation of lymphocytes [11]. On the recipient side, a greater risk of chronic graft-versus-host disease to G-CSF stimulated PBSCT vs. bone marrow-derived grafts has been reported [12]. Consequently, significant activity has been dedicated to the identification and development of alternative mobilizing agents that would combine predictable, efficient stem cell mobilization with single dose activity and good tolerability for both donor and recipient.

The ability of CXCR4 antagonists to rapidly dislodge stem cells from the marrow has been recognized for many years [13]. Previously studied compounds were neither universally well tolerated nor very potent [14]. The only currently approved CXCR4 antagonist plerixafor mobilized a median of 16 CD34+ cells per µL when administered alone (without G-CSF) in healthy donors [15], which is barely sufficient to generate a normal-sized graft in two apheresis sessions.

The synthetic protein epitope mimetic (PEM) peptidic CXCR4 antagonist POL5551, a close analogue of the clinical stage compound balixafortide (POL6326), shows a very wide pharmacodynamic range in preclinical models and at optimal doses even mobilizes more efficiently than G-CSF [16]. In this clinical phase I dose escalation trial we evaluated balixafortide with regard to its safety, tolerability, pharmacokinetics and mobilization efficiency in healthy male volunteers in comparison to G-CSF. Balixafortide was well tolerated and rated subjectively preferable to G-CSF according to the volunteers. Mobilization was rapid; dose-dependency was apparent at doses up to 1500 µg/kg with an average peak mobilization of 38.2 ± 2.8 CD34 positive cells per µL.

## Methods

### Volunteers

Volunteers were healthy male HSC donors from the German Stem Cell Donor Registry (DSSD) who had received a 5-day course of filgrastim (G-CSF, 7.5–10 µg/kg per day in 2 divided doses) for matched-unrelated stem cell donation and shown a grossly average mobilization response (121.6 ± 8.6 CD34+ cells/μL). Additional eligibility (inclusion) criteria for treatment with balixafortide were the same as for G-CSF mobilized stem cell donation [11]. Between G-CSF mobilization/HSPC donation and study participation there was a wash-out period of at least 6 weeks.

Written informed consent was provided prior to performing any study related activities. The study and all related documents were approved by the local Institutional Review Board (IRB) (#324/11) and the federal medicines agency BfArM (approval #61-3910-4037635). The trial was registered with the European Medicines Agency as EudraCT-Nr. 2011-003316-23 and on *clinicaltrials.gov* as NCT01841476.

Study drug was administered on an in-patient basis in the phase I clinical trial unit of Goethe University Medical Center, the ‘Klinisches Studienzentrum Rhein-Main’. Volunteers were discharged 24 h after treatment, to return for a follow-up appointment 8–14 days thereafter.

### Study design

This was a prospective Phase I open label dose escalation trial; The study design is summarized in Table 1. A total of 27 volunteers were treated with balixafortide. A treatment consisted of a single intravenous infusion of balixafortide in normal saline at doses of 500, 1000, 1500, 2000 and 2500 μg/kg, based on actual weight, followed by sequential clinical and blood analyses (see below). Initially conceived as a classical 3 + 3 dose escalation design, the volunteers were assigned to four groups defined by increasing dose levels of balixafortide (500, 1000, 1500, and 2000 μg/kg) administered by constant rate infusion at over 2 h. Subsequently, amendments were added to test additional modalities: Group 6 received 2500 μg/kg under the same conditions. Volunteers assigned to Group 5 received a dose level of 2000 μg/kg by an continuously increasing infusion rate (ramp-infusion instead of constant rate infusion) applied over 2 h. In group 7, a dose level of 1000 μg/kg was infused over 1 h at a constant rate and compared (intra-individually) to the 2 h infusion given with an interval of ≥4 weeks. A second balixafortide treatment was furthermore tested in volunteers from groups 2, 3 and 6 with groups 2 and 3 receiving 2500 μg/kg and group 6 given 1500 μg/kg as the second infusion. In as far as not all volunteers from the initial phase of the study could be recalled, they were replaced by new volunteers receiving two treatments, to have a group size of at least 3 for each cross-over modality, explaining the variable dosing group sizes between 3 and 6 (Table 1). Thus, to allow for intra-individual comparison, 12 donors received a second dose of balixafortide (2 h constant infusion rate for all) after a minimum wash-out period of 4 weeks.

**Table 1:** Study design

	1st treatment Dose (μg/kg)/infusion time (h)/infusion rate	2nd treatment Dose (μg/kg)/infusion time (h)/infusion rate
Group 1		
Volunteer 1	500/2/constant	_
Volunteer 2	*500/2/constant*	
Volunteer 3	500/2/constant	
Group 2		
Volunteer 1	1000/2/constant	*2500/2/constant* ^H^
Volunteer 2	1000/2/constant	*2500/2/constant* ^H^
Volunteer 3	*1000/2/constant*	–
Volunteer 4	*1000/2/constant*	*2500/2/constant* ^H^
Group 3		
Volunteer 1	*1000/1/constant* ^h^	*1000/2/const. rate*
Volunteer 2	1000/1/constant	1000/2/const. rate
Volunteer-3	1000/1/constant	1000/2/const. rate
Group 4		
Volunteer 1	1500/2/constant	*2500/2/const. rate* ^H^
Volunteer 2	*1500/2/constant* ^h^	*2500/2/const. rate* ^H^
Volunteer 3	1500/2/constant	–
Volunteer 4	1500/2/constant	*2500/2/const. rate* ^H^
Group 5		
Volunteer 1	*2000/2/constant* ^h^	–
Volunteer 2	*2000/2/constant*	
Volunteer 3	*2000/2/constant* ^h^	
Volunteer 4	*2000/2/constant* ^h^	
Volunteer 5	*2000/2/constant* ^h^	
Volunteer 6	*2000/2/constant* ^h^	
Group 6		
Volunteer 1	2000/2/ramped	–
Volunteer 2	*2000/2/ramped* ^h^	
Volunteer 3	*2000/2/ramped*	
Group 7		
Volunteer 1	*2500/2/constant* ^H^	–
Volunteer 2	2500/2/constant^H^	1500/2/const. rate
Volunteer 3	2500/2/constant^H^	*1500/2/const. rate*
Volunteer 4	2500/2/constant^H^	1500/2/const. rate

A total of 27 volunteers were treated with 39 doses of balixafortide

In italics volunteers with histamine release associated AE

^*H*^Anti-histamine premedication

^*h*^ *T*herapeutic anti-histamine treatment upon appearance of likely histamine release AEs

Vital signs were monitored immediately prior to and in the first 24 h after the start of the infusion of balixafortide; serial blood samples were drawn for biochemical safety profiling and pharmacokinetic/pharmacodynamic analyses. Given the cationic nature of the compound [17] the risk of local or systemic symptoms of histamine release was identified and anti-histamine treatment was proposed (per protocol) in case of such symptoms. After completion of the 2000 µg/kg dosing group the protocol was amended to introduce prophylactic anti-histamine treatment in the dosage group ≥2500 µg/kg. Volunteers who received prophylactic or therapeutic anti-histamine medication are listed accordingly in Table 1.

### Objectives

Primary outcome parameters were safety and tolerability of balixafortide when compared to G-CSF, pharmacodynamics of mature and immature blood cell mobilization, specifically the intra-individual comparison of balixafortide- and G-CSF-induced mobilization of HSPCs. Secondary objectives included pharmacokinetic analyses and identification of a suitable window for HSPC apheresis.

### Pharmacokinetics

Plasma samples were collected at the indicated times and kept frozen until immediately before analysis.

### Pharmacodynamics

**Fig. 1:**
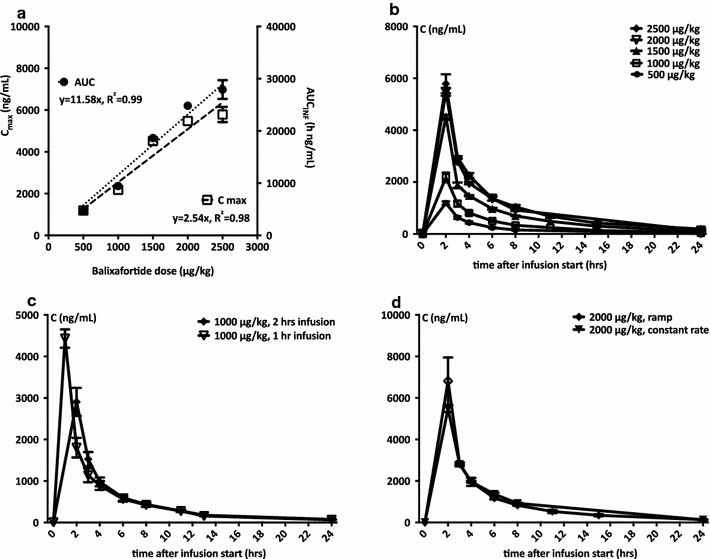
Pharmacokinetics. **a** C_max_ (*left Y-axis*) and AUC (*right Y-axis*) are plotted as a function of dose (*X-axis*). Dose-linear pharmacokinetics were observed. n = 3–6. **b** Dose-dependent pharmacokinetic profiles are shown. C_max_ was reached at the end of infusion and balixafortide was cleared quickly from the circulation thereafter. n = 3–6. **c** Comparative pharmacokinetics of balixafortide (1000 µg/kg) infused over 1 vs. 2 h. C_max_ was higher and reached earlier for 1-h dosing, but AUC was similar (data not shown). n = 3. **d** Comparative pharmacokinetics of linear vs. ramped infusion rate at the 2000 µg/kg dose level. C_max_ was notably higher but AUC was virtually identical for both infusion types. n = 3. Mean ± SEM are shown throughout

**Fig. 2:**
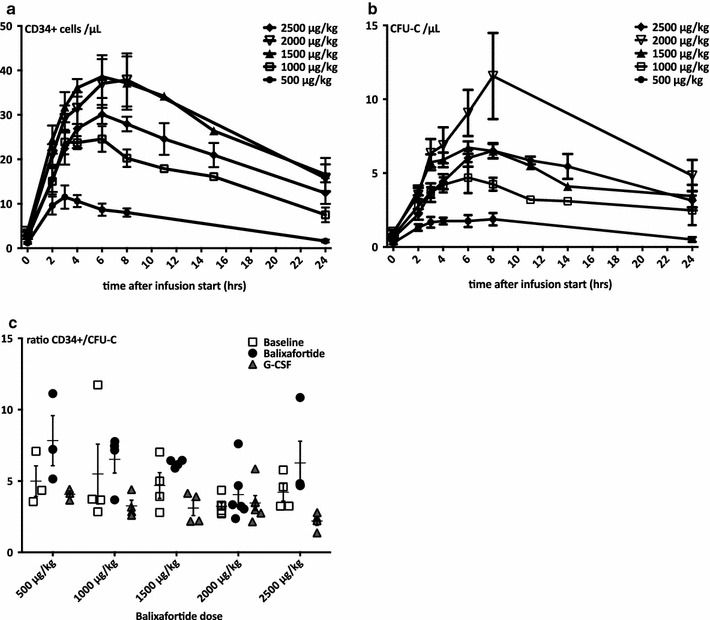
Pharmacodynamics: mobilization of immature hematopoietic cells. **a**, **b** Dose-dependent mobilization of phenotypically (CD34+ , *panel* **a**) or functionally (CFU-C, *panel* **b**) defined stem and progenitor cells (HSPCs). HSPC mobilization was observed at all dose levels. Mobilization at the lowest dose level peaked 1 h after the end of the infusion and was delayed after higher doses. Dose dependence was observed for the first three dosing steps (n = 3–6, mean ± SEM).** c** The ratio between circulating CD34+ cells and CFU-C is shown for all doses. Clonogenicity of balixafortide mobilized CD34+ tended to be lower than for G-CSF mobilized CD34+ cells. *Symbols* represent individual values, the *short horizontal bar* and *whiskers* mean ± SEM. n = 3–6

**Fig. 3:**
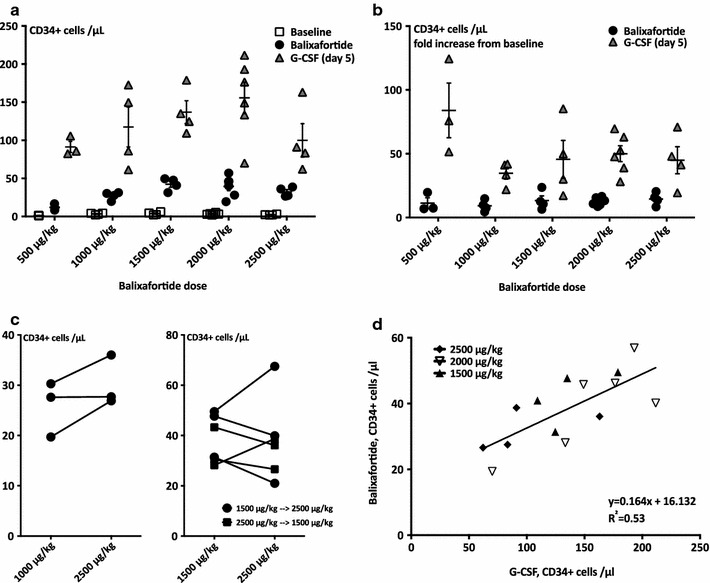
Mobilization of immature hematopoietic cells: comparison with G-CSF. Baseline circulating CD34+ cells, balixafortide mobilized CD34+ cells (incremental balixafortide dose as indicated on* X-axis*) and G-CSF mobilized CD34+ cells (same G-CSF dose for all groups, see “Methods” section) are displayed in (**a**). Corresponding fold-increase data are shown in (**b**). *Symbols* represent individual values, the *short horizontal bar* and *whiskers* mean ± SEM. n = 3–6. **c** Intra-individual comparison of peak mobilization with 1000 vs. 2500 µg/kg (*left panel*, n = 3) or 1500 vs. 2500 µg/kg (*right panel*, n = 6) of balixafortide is shown. 2500 µg/kg balixafortide mobilized more CD34+ cells than 1000 µg/kg, whereas no difference between peak mobilization with 1500 vs. 2500 µg/kg was observed. *Symbols* represent individual values. **d** The good correlation between effectiveness of G-CSF vs. balixafortide with respect to CD34+ cell mobilization is displayed for balixafortide doses between 1500 and 2500 µg/kg n = 14. *Symbols* represent individual values (constant rate only)

**Fig. 4:**
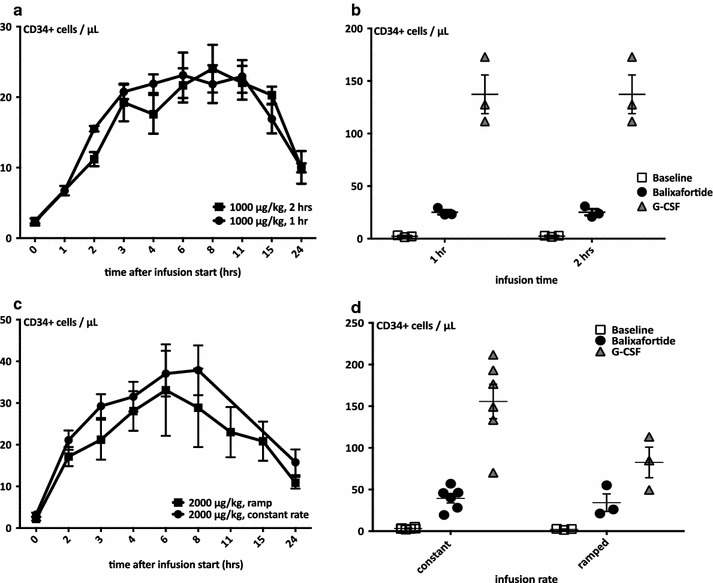
Mobilization of immature hematopoietic cells: effects of infusion velocity and rate. **a**, **b** Inter-individual comparison of CD34+ cell mobilization after infusion of balixafortide over 1 vs. 2 h demonstrates equivalent mobilization kinetics (**a**) and peak mobilization responses (**b**). This cohort received only a single course of G-CSF, hence the data shown for G-CSF mobilization with each of the baselines and balixafortide treatments are the same. *Symbols* represent individual values, the *short horizontal bar* and *whiskers* mean ± SEM. n = 3. **c**, **d** Comparison of CD34+ cell mobilization after ramped vs. constant rate infusion of balixafortide demonstrates equivalent mobilization kinetics (**c**) and peak mobilization responses (**d**). *Symbols* represent individual values, the *short horizontal bar* and *whiskers* mean ± SEM. n = 3

**Fig. 5:**
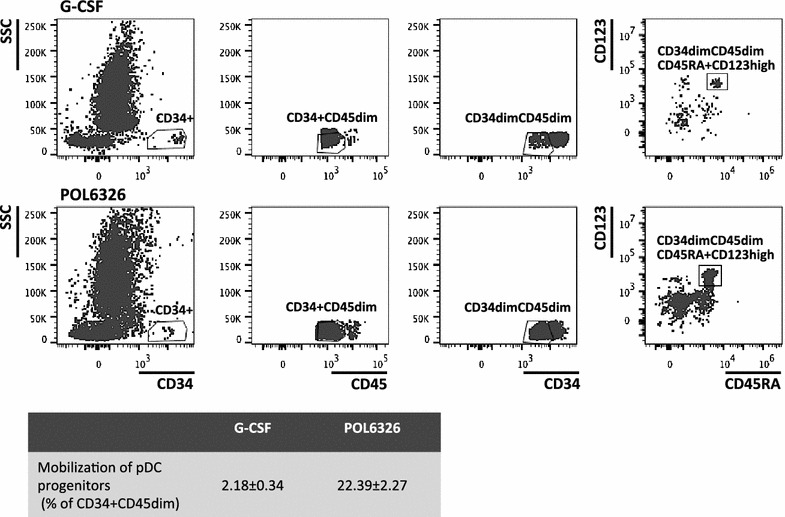
Mobilization of plasmacytoid dendritic cell (pDC) progenitors. Displayed are representative flow cytograms of putative pro-pDCs (defined as CD34^dim^CD45^dim^CD45RA^+^CD123^high^) detected in G-CSF (*top*) or balixafortide (*bottom*) mobilized blood. Mean (±SEM) percentages of pDC progenitors within the HSPC fraction CD34^+^CD45^dim^ detected in all G-CSF and balixafortide (1st treatment, constant infusion rate, 2 h, Table 1) mobilized specimen (n = 21) are shown in the *bottom*

**Fig. 6:**
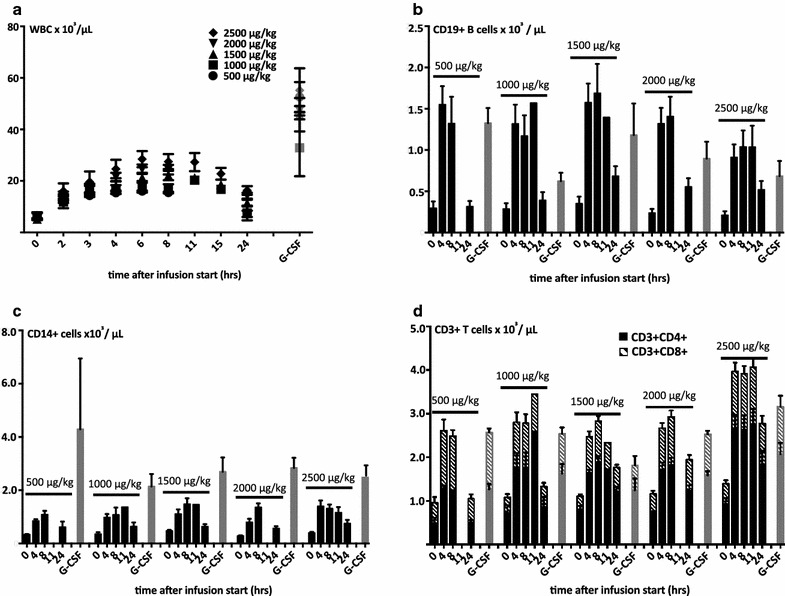
Pharmacodynamics: mobilization of mature hematopoietic cells. **a** Balixafortide induces dose-dependent leukocytosis with the same kinetics as observed for CD34+ cells (n = 3–6, mean ± SEM); -fold difference between mature cell mobilization with balixafortide and G-CSF was equivalent to that for CD34+ cells. Differential mobilization of leukocyte subsets was observed, with lower monocyte (**b**), higher B-cell (**c**), but similar T-cell (total, CD4+ and CD8+ T-cells, **d** mobilization after balixafortide vs. G-CSF induced mobilization (n = 3–6, mean ± SEM). G-CSF was administered at the same dose in all groups; the symbol used links mobilization results to a certain balixafortide dosing group

Blood samples were collected at the indicated times (Figs. 1, 2, 3, 4, 5 and 6) and kept at room temperature (maximum 2–3 h.) until immediately before analysis. Complete blood counts were assessed with the Sysmex XT1800 hematology analyzer (Norderstedt, Germany). CD34+ cells were quantified using the single platform flow cytometry analysis with the SCE Kit [Becton–Dickinson (BD), Heidelberg, Germany] according to the manufacturer’s instructions and ISHAGE guidelines [18]. In addition, multi-parametric flow cytometric analyses were performed to quantify co-mobilized mature cell subsets such as T (CD45+ CD3+), B (CD45+ CD19+) and NK (CD45+ CD56+ 16+) cells (Multitest, T cells, BD), T cell subpopulations (CD45+ CD3+ CD4+/CD8+, Multitest, TBNL cells, BD) and monocytes (CD45+ CD14+, all from BD). In addition, plasmacytoid dendritic cell progenitors (pro-pDCs) were identified as CD45dimCD34dimCD45RA + CD123high (all moABs from BD). Lyse-no-wash protocols were used in conjunction with BD counting beads for direct cell enumeration for CD34+ cells and T cell subsets; all other cell concentrations were calculated using frequencies relative to directly enumerated cell species, such as CD34+, CD45+ or CD3+ cells.

Circulating colony-forming units-culture (CFU-C) were quantified by plating aliquots of lysed peripheral blood in commercial cytokine-replete methylcellulose media (StemMACS HSC-CFU lite with Epo, human, Miltenyi Biotec GmbH, Bergisch-Gladbach, Germany).

### Statistical analysis

Unless otherwise mentioned, data are expressed as mean ± SEM. Descriptive statistics and Student’s t-test for paired or unpaired analysis (as appropriate) were calculated using Excel. A p < 0.05, Bonferroni-corrected for multiple testing if appropriate, was considered statistically significant.

## Results

### Safety and tolerability

A summary of adverse events that were documented throughout the trial is shown in Table 2. No severe adverse events (SAEs) were observed. Mild skin reactions such as flushing, urticaria or local itching were reported by 1/3, 4/10, 2/7, 8/9 and 7/10 volunteers receiving 500, 1000, 1500, 2000 and 2500 µg/kg of balixafortide respectively. Upon treatment with a combination of H1 and H2 blockers symptoms rapidly abated. These reactions were rated likely related to study drug.

**Table 2:** Safety and tolerability of balixafortide. Summary of adverse events

Dose (μg/kg)	500	1000	1000	1500	2000	2000	2500
Infusion time/rate	2 h./const.	2 h./const.	1 h./const.	2 h./const.	2 h./const.	2 h./ramp	2 h./const.
n Volunteers	3	7	3	7	6	3	10
Volunteers with AE	1	5	1	2	6	2	7
Erythema				1	4		7
Pruritus		2	1	1	4	2	
Infusion site erythema	1			2			
Infusion site pruritus		1		1			
Urticaria				1	1		
Flushing					1	1	
Hypoasthesia oral							2
BP increase		2					
Blood CK increased					2		
Infusion site irritation				1			
Feeling hot							1
Muscle tightness							1
Headache							1
Hypoasthesia					1		1
Throat tightness							1
Bone pain					1		
Vertigo							1

A total of 27 volunteers were treated with 39 doses of balixafortide. Therefore some volunteers (12) were included in two different groups, when adverse events per dose-group were assessed. Responses to all items were binary (yes/no), not quantitative; multiple responses were possible

Three adverse events (AEs) were considered possibly related to study drug: mild bone pain (1 subject), an unexplained elevation in serum creatinine kinase (2 subjects), and a systolic blood pressure reading of >150 mmHg (2 subjects).

Constant-slope infusion, tested at the 2000 µg/kg dose level in three volunteers (group 6), as well as increased infusion rates (1 vs. 2 h, group 3), tested in paired analyses in three volunteers at the 1000 µg/kg dose level, did not influence the tolerability of the agent (Table 1).

At the time of follow-up, volunteers were questioned about their subjective rating of G-CSF vs. balixafortide as mobilizing agents; there was an overwhelming preference for balixafortide. See also Table 3 for the questionnaire and volunteer responses.

**Table 3:** Safety and tolerability of balixafortide. Subjective rating

Side effects in the course of treatment with:	G-CSF	Balixafortide
Abdominal pain	2	0
Bone pain	24	0
Headache	11	1
Skin reactions	1	13
Flu-like symptoms	21	0
Vomiting	0	1
Palpitation	1	0
Fever	2	0
Sweating	0	1
Fatigue	11	4
Insomnia	0	1
The treatment is		
Easy to use	7	29
Acceptable	13	30
More convenient overall	2	32

### Pharmacokinetics

Serial plasma samples were assayed for balixafortide concentrations and pharmacokinetic parameters were calculated using Phoenix WinNonlin 6.4. We observed dose linearity for both Cmax and AUC (Fig. 1a). The volume of distribution was approximately 500–600 mL/kg. Balixafortide was cleared from plasma with a terminal half-life of approximately 5 h over all application schemes and doses of 5:45 ± 0:35 h (mean ± SD; Fig. 1b). The clearance of balixafortide appeared to be almost equal to the glomerular filtration rate suggesting that balixafortide is mainly cleared through the kidney. Different infusion durations (1 vs. 2 h) did not notably influence the PK profile except for an earlier C_max_ (Fig. 1c), and the same applied to ‘constant-slope’ vs. ‘ramp’ infusion (Fig. 1d).

### Pharmacodynamics—mobilization of immature cells

At all doses tested, balixafortide infusions quickly resulted in an increase in circulating HSPCs, as measured phenotypically (CD34+ cells, Fig. 2a) or functionally in colony assays (Fig. 2b). Clonogenicity of balixafortide vs. G-CSF mobilized CD34+ cells was lower with 1 CFU-C out of 5.9 ± 0.5 balixafortide mobilized CD34+ cells vs. 1 CFU-C out of 3.2 ± 0.2 CD34+ cells mobilized with G-CSF (Fig. 2c). At lower doses (500 vs. 1000 vs. 1500 µg/kg), dose-dependent mobilization was clearly observed, while the later dose increments to 2000 and 2500 µg/kg did not result in a commensurate increase in the number of mobilized HSPC compared to 1500 µg/kg (Fig. 3a, b). This was confirmed in paired analyses in small cohorts (Fig. 3c). Therefore, for some analyses all mobilization data for doses ≥1500 µg/kg are analyzed together. As such, mean peak mobilization in response to doses of 1500–2500 µg/kg was 38.2 ± 2.8 CD34+ cells/µL (Fig. 3d). Thus at these doses intra-individual comparison of balixafortide vs. G-CSF induced mobilization revealed that—on average—the G-CSF regimen was about three times as effective as the CXCR4 antagonist. There appeared to be a good correlation between the two mobilizing agents (Fig. 3d), suggesting that—as had been shown in mice [16, 19]—good mobilizers mobilize efficiently with either agent and poor mobilizers are refractory to both.

Peak mobilization at the 500 µg/kg dose was observed 1 h after the end of the balixafortide infusion/after reaching C_max_ (Figs. 1b, 2a). At higher doses, the observed mobilization peak appeared later, approximately 4 h after the end of the infusion. Thereafter, the number of circulating CD34+ cells slowly decreased but remained elevated beyond baseline at the 24 h time point for all except the lowest dose (Fig. 2a, b). Constant-slope (ramp) vs. constant-rate infusions (at 2000 µg/kg only) had no discernible effect on stem cell mobilization efficiency, and the same applied to infusion rate (1 vs. 2 h) (Fig. 4).

A population of “stem cells” co-expressing CD45RA and CD123, previously described in blood of plerixafor-mobilized donors and identified as plasmacytoid dendritic cell progenitors (pro-pDCs) [20], was detected at high frequencies (22.4 ± 2.3% of SSCdim/FSCmid-hi/CD45dim/CD34+ cells) after balixafortide-treatment, but was rare after G-CSF (Fig. 5).

### Pharmacodynamics—mobilization of mature hematopoietic cells

Stem cell mobilization was accompanied with mixed leukocytosis affecting all cell lineages. It followed the same kinetics as stem cell mobilization and was dose-dependent as well as short-lived. At balixafortide doses of 1500–2500 µg/kg white blood counts (WBCs) of 25.3 ± 1.4 × 10*3 WBC/µL were reached, i.e. balixafortide mobilized approximately half as many mature cells as G-CSF (Fig. 6a). The lineage distribution of mature leukocytes differed markedly between both agents, in that balixafortide mobilized higher relative and absolute numbers of B-cells and fewer myeloid cells (Fig. 6b, c). The ratio between T-lymphocytes and CD34+ cells was 26.2 ± 1.98:1 in G-CSF mobilized blood, vs. 95.7 ± 8.9:1 in balixafortide mobilized blood, predicting that apheresis products from balixafortide mobilized donors will contain more T-cells than from G-CSF treated donors. Within the T cell population the proportion of T helper (CD4+) and cytotoxic T cells (CD8+) was very similar between the differently mobilized blood specimens as well as compared to steady state (baseline) (Fig. 6d).

## Discussion

We performed a Phase I clinical trial to directly compare the novel CXCR4 antagonist balixafortide with the standard mobilizing agent G-CSF, with regard to the following parameters: safety and tolerability, pharmacokinetic profile, and pharmacodynamic effects; the latter were defined as mobilization volumes of immature and mature blood cells. At the doses tested, balixafortide was associated with few adverse effects, and none of these were dose limiting. Skin symptoms compatible with possible local histamine release syndrome were observed with some regularity but could easily be managed with routine co-administration of anti-histamines.

Mechanistically, mobilization with CXCR4 antagonists like balixafortide involves a rather short-lived interference with stem cell retention in the bone marrow [13]. Therefore some of the proposed (albeit never robustly substantiated) long-term adverse effects of G-CSF should not rationally be associated with balixafortide treatment. Specifically, we speculate that, unlike G-CSF, balixafortide may be a safe mobilizing agent for patients (and donors) with autoimmune conditions [21, 22] as well as sickle cell disease [23, 24]. Formal questionnaires also confirm good tolerability and, in fact, indicate a preference for balixafortide vs. GCSF, although the study setting may have favored balixafortide.

The higher balixafortide doses tested in this study mobilized 38.2 ± 2.8 CD34+ cells/µL. In view of the currently available technologies [25, 26] this is sufficient to generate a stem cell product with an average of 5× 10E6 CD34 + donor cells/kg as per a single apheresis; this constitutes an adequate number of cells for the average PBSCT [i.e. >4× 10E6/kg (weight of recipient), and would even accommodate patients with a body weight that is somewhat higher than their donor. That said, data in mice [16] and cynomolgus monkeys (unpublished) indicated a semi-logarithmic dose-response relationship with a high ceiling; extrapolating from these data we postulate that further dose increments of balixafortide are possible. Further studies with higher doses of balixafortide are therefore warranted as meaningfully higher stem cell yields may be achieved—provided these doses are well tolerated.

The comparator agent in our study was G-CSF given in split doses as is routine practice in our center [7]; the rationale is twofold: more efficient mobilization and potentially better tolerability [27–30]. The alternative regimen that is widely used in the USA employs the same daily dose but makes use of a single injection for which average CD34+ cell counts in the mid-sixties’ range (per µL) were reported [6] i.e. less than two-thirds of what is achieved with split-dose G-CSF. Thus the advantage in efficiency of single-dose G-CSF vs. balixafortide at the doses tested here would be less than twofold.

High frequencies of a population presumed to represent precursors of plasmacytoid dendritic cells (pro-pDCs) were previously detected in plerixafor-mobilized blood [20] and were also found by us in balixafortide-mobilized blood (Fig. 5). This indicates a substance class specific mobilization effect and is in fact in line with reports showing the importance of the CXCR4/CXCL12 pathway in pro-pDC development (and retention) in mice [31]. The biological function of pro-pDCs in a graft is unclear. As they have been associated with immunomodulatory functions [32] such as promotion of regulatory T cell differentiation [33, 34], it is tempting to speculate about a possible role in modulating graft-versus-host disease (GvHD). Indeed, in a cohort of patients receiving a plerixafor-mobilized graft only 1/20 developed acute GvHD 3° or 4° [15] which is markedly less than would be expected with G-CSF-mobilized blood [12, 35, 36] and which was not accompanied by an excessive relapse rate. These data are potentially meaningful given the much higher T-cell dose co-transplanted with a CXCR4 antagonist-mobilized graft.

As all other mobilizing regimes, mobilization by balixafortide was associated with marked leukocytosis; the-fold difference for mature and immature cell mobilization between G-CSF and balixafortide was quite similar. However, the distribution of leukocyte subtypes was markedly different; specifically the virtually diagnostic left-shifted neutrophilia in G-CSF treated volunteers [2, 5] was not observed after balixafortide. These observations support the prediction that balixafortide mobilizes without stimulation and lineage skewing and might thus be suitable for patients in whom such could result in undesirable side effects.

One of the desired features of a mobilizing agent is predictability of efficacy. Mouse data clearly indicate that mobilization efficiency is dominated by genetics [37, 38] but that the delta or-fold difference (e.g. between C57Bl/6 and DBA/2 mice) is much closer for CXCR4 antagonists than for G-CSF [16]. Although differences in G-CSF mobilization efficiency in our trial were less apparent due to inclusion criteria (average mobilization), our data clearly confirm the strong donor-inherent component for the efficiency of stem cell mobilization per se on the one hand and less pronounced variability in mobilization response with balixafortide on the other hand.

## Conclusion

We have demonstrated that HSPC mobilization with balixafortide can be both efficient and predictable; more potent mobilization may be achievable with higher doses of this agent, as future studies may be able to show. Balixafortide treatment was safe and well tolerated. Because of its mechanism of action along with its rapid elimination, this stem cell mobilizing agent can be considered an option for many of the patients and donors with contra-indications to G-CSF. Its brisk mobilization after one single dose is also highly convenient for donors and apheresis centers. In aggregate, balixafortide could be developed as an alternative single-agent mobilizing agent for patients and donors alike. By extension, our work also demonstrates the potential of PEM technology for rational drug design.
